# Incidence and clinicopathologic behavior of uterine cervical carcinoma in renal transplant recipients

**DOI:** 10.1186/1477-7819-9-72

**Published:** 2011-07-13

**Authors:** Sung Taek Park, Min Jong Song, Jong Sup Park, Soo Young Hur, Chung Won Lee

**Affiliations:** 1 Department of Obstetrics and Gynecology, Seoul St. Mary's Hospital, The Catholic University of Korea, Seoul, Republic of Korea; 2 Department of Obstetrics and Gynecology, Daejeon St. Mary's Hospital, The Catholic University of Korea, Seoul, Republic of Korea; 3 Department of Obstetrics and Gynecology, Yeouido St. Mary's Hospital, The Catholic University of Korea, Seoul, Republic of Korea

**Keywords:** Cervical carcinoma, Renal transplantation, Human papillomavirus, Clinicopathologic behavior

## Abstract

**Background:**

Renal allograft recipients are reported to have a higher incidence of malignancy than the general population. This single hospital-based study examined the incidence and clinicopathologic behavior of uterine cervical carcinoma in renal transplant recipients.

**Methods:**

Among 453 women receiving renal transplantation from January 1990 to December 2008, 5 patients were diagnosed with cervical carcinoma. Medical records of these 5 patients were retrospectively reviewed, and clinicopathologic data were collected and analyzed.

**Results:**

The incidence of cervical carcinoma in renal transplant recipients was 58.1 out of 100,000 per year, which is 3.5 times higher than in the general Korean population. The mean interval between the time of renal transplantation and the time of cervical carcinoma diagnosis was 80.7 months. After a median follow-up of 96.2 months, there was no recurrence of the disease or death. In 4 patients who were positive from human papillomavirus in situ hybridization (HPV ISH), high or probably high risk HPV DNA was detected in all. Punctate staining of HPV ISH was detected in 3 out of 4 patients.

**Conclusions:**

Higher incidence of cervical carcinoma is expected in renal transplant recipients, so appropriate surveillance is needed to ensure early detection and treatment of cervical carcinoma.

## Background

Recent advancements in immunosuppressive therapy have reduced cases of acute rejection and improved the long-term survival rate of grafts in organ transplant patients. Long-term use of immunosuppressants has likewise increased the incidence of infectious diseases, autoimmune diseases, and malignancy in organ transplant patients.

Particularly, the incidence of malignancy was reported to be 2-31% in patients who had received renal transplants [1-6], and was also reported to be a major cause of one-third of cases of post-organ transplant death [7]. Possible mechanisms by which immunosuppressants increase malignant carcinoma include the increased chances of infection with such oncogenic viruses as the human papillomavirus (HPV), DNA damage due to the immunosuppresant, increased carcinogenicity of carcinogens due to the immunosuppressant, and immunologic tolerance to oncocyte due to immunosuppresion [8,9].

HPV is known as the major cause of cervical carcinoma and cervical intraepithelial neoplasia (CIN)[10,11]. More than 100 types of HPV are known, and among these, 15 types are classified as high-risk HPV. Generally, most HPV infections do not have symptoms and disappear within 12 months of the onset of infection, although high-risk HPV has been reported to be related to persistent infection and the onset of CIN [12].

In the early stage of the developmental course of CIN to invasive carcinoma, the phase at which the HPV DNA integrates into the host's DNA has been known to play an important role, although such developmental course has yet to be clearly identified. It was reported that the E2 gene loses its regulating function through this process, and that subsequent over-expression of the E6 and E7 genes causes loss of control over the cell cycle. Taking these into account, the accumulation of variations of multiple genes causes invasive cervical carcinoma [13].

Recent studies reported the usefulness of In Situ Hybridization (ISH) for HPV, a method of testing whether or not the HPV DNA, which has an important role in early oncogenesis, is integrated into the host's DNA. It was also reported that when the HPV DNA integrated into the host's DNA, a punctate pattern occurred in which obvious round spots were observable in the nucleus. When unitegrated, the episomal HPV DNA existed, and a diffuse pattern was observable in which the nucleus was uniformly stained [13].

As described, immunosuppresants used after a renal transplant to reduce the rejection reaction to graft may increase the chances of opportunistic infection with HPV, and the immunosuppressive condition itself may also increase the risk of cervical carcinoma through immunologic tolerance to oncocytes with accumulated genetic variations [8,9].

In South Korea, statistical data on a national level on the incidence of malignancy in patients who had received a renal transplant are not available, and no proper screening method for these patients has been established. As such, this study was performed to investigate the incidence of cervical carcinoma and other malignant carcinomas in female patients who had received a renal transplant at a single hospital, to analyze the clinicopathological features of patients with cervical cancer and to analyze whether or not the HPV DNA integrated into the host's DNA with the use of ISH.

## Methods

This study was conducted according to the Declaration of Helsinki, and approval was obtained from the Internal Review Board for the clinical trial. A total of 453 female patients who had received a renal transplant from January 1990 to December 2008 at Kangnam St. Mary's Hospital of Catholic University were included in the study. According to a retrospective review of the medical charts of these patients, 36 of them had malignancy. The incidence of carcinoma was analyzed according to the type of carcinoma. The clinical factors related to the renal transplant and the clinicopathological factors related to cervical carcinoma were analyzed based on the medical records of the five patients who were diagnosed with cervical carcinoma. In addition, for the HPV ISH and the analysis of the HPV type, a TMA core with a size of 5 mm, which is sufficient for observation, was prepared using the specimen from the uterine cervix that was removed through hysterectomy. Using the TMA of the uterine cervix, a 4 mm-thick slice was prepared and attached to the coated slide, and then used for the ISH [13].

### HPV ISH of the uterine cervical tissue

**Figure 1:**
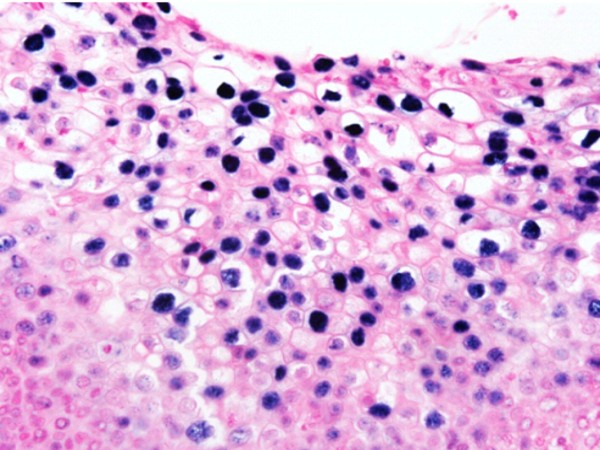
**HPV ISH**. Diffuse signal pattern (H&E, × 400). presents the ISH signals of HPV shown by Inform HPV III kit (Ventana Medical System). The diffuse signal pattern indicated that signals are condensed and uniformly packed in the nucleus.

**Figure 2:**
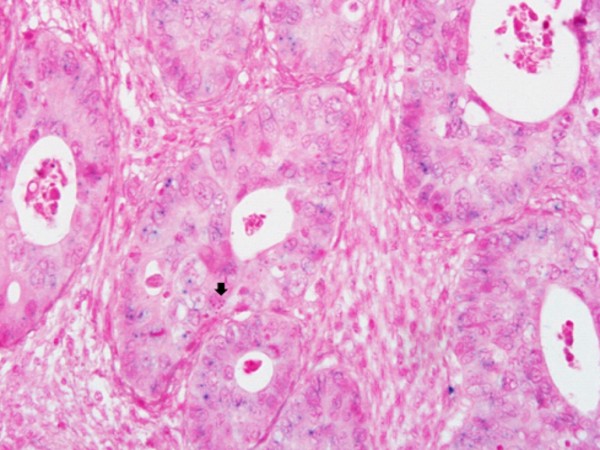
**HPV ISH**. Punctate signal pattern (H&E, × 400). presents the ISH signals of HPV shown by Inform HPV III kit (Ventana Medical System). The punctate signal pattern indicated that signals are dot-like and sparsely distributed in the nucleus.

The Inform HPV III (Ventana, Tucson, AZ, USA) probe that can identify 12 types of high-risk HPV(16, 18, 31, 33, 35, 39, 45, 51, 52, 56, 58, and 66) was used. The HPV ISH was performed using the BenchMark Automated Slide Staining System (Ventana Medical System) according to the manufacturer's instructions. The result of the nuclear staining was interpreted as positive, and the pattern of the nuclear staining was classified as diffuse, punctated, and mixed [13]. "Diffuse pattern" was defined as a pattern in which the nucleus was diffusely but strongly stained (Figure 1); and "punctated pattern," as a pattern in which the nucleus was stained, as if the nucleus was marked with several obvious round dots (Figure 2).

### HPV typing

The paraffin block, in which the patient's tissue was stored, was deparaffined, and the DNA was extracted [14]. Subsequently, HPV typing was performed using the commercial GoodgeneHPVchip (Goodgene Inc., Seoul, Korea), an HPV DNA chip, according to the manufacturer's instructions.

In brief, this genotyping was for a microarray system that included probes of 40 types of HPV [14 types of high-risk HPV (16, 18, 31, 33, 35, 39, 45, 51, 52, 56, 58, 59, 68, and 82), seven types of moderate or probably high-risk HPV (26, 53, 66, 69, 70, and 73), 13 types of low-risk HPV (6, 11, 34, 40, 42, 43, 44, 54, 55, 61, 62, 72, and 81), and nine other types of HPV (7, 10, 27, 30, 32, 57, 83, 84, and 91)][15]. The detection of one or more of these types of HPV was expressed as positive.

## Results

### Incidence of malignancy after a renal transplant

A total of 453 female patients had received a renal transplant from January 1990 to December 2008 at Kangnam St. Mary's Hospital. Of these, 36 patients (7.9%) were found to have had malignancy during the follow-up. The most common type of malignancy was thyroid carcinoma followed by cervical carcinoma and bladder carcinoma. (Table 1).

**Table 1:** Clinical manifestations of study population (1990-2008)

Type of malignancy	**No**.	Incidence (%)
Thyroid carcinoma	7	1.6
Cervical carcinoma	5	1.1
Bladder carcinoma	5	1.1
Breast carcinoma	5	1.1
Malignant lymphoma	4	1.0
Hepatocellular carcinoma	2	0.4
Colorectal carcinoma	2	0.4
Non-melanocytic skin carcinoma	2	0.4
Kaposi's sarcoma	1	0.2
Stomach carcinoma	1	0.2
Parathyroid carcinoma	1	0.2
Malignant thymoma	1	0.2
Total	36	7.9

### Clinicopathological analysis of cervical carcinoma that developed after a renal transplant

The mean age at the time of the renal transplant was 30.8 years (25-41 years). Three of the five patients used Cyclosporin as an immunosuppressant, and two switched to Tacrolimus due to their acute rejection reaction. The grafts were well-maintained in four of the five patients, whereas in one patient, the graft lost its function, which led to hemodialysis.

The mean period of the dialysis before the renal transplant was 13.4 months (1-51 months) (Table 2). The mean age at which a patient was diagnosed with cervical carcinoma was 37.4 years (27-41 years). Thus, the time of diagnosis of the cervical carcinoma from the renal transplant was 80.7 months on the average (5.6-136.8 months). Three patients were diagnosed with squamous cell carcinoma; one patient, with adenosquamous cell carcinoma; and one patient, with adenocarcinoma. All of them received surgical treatment. One patient with la1 cervical carcinoma underwent extrafascial hysterectomy without node dissection; two patients with lb1 and two patients with lb2, type III radical hysterectomy with pelvic lymphadenectomy. In the four patients who underwent pelvic lymphadenectomy, lymph node metastasis was not observed, and no additional chemotherapy or radiation therapy was performed. The mean follow-up period after the diagnosis of the uterine cervical carcinoma was 93.2 months (18-190 months), during which no death o or recurrence of disease occurred (Table 3).

**Table 2:** Clinicopathologic characteristics of cervical cancer patients

Case No.	Age at the time of KT (years)	Original kidney disease	Pre-KT treatment (Duration, months)	Maintenance immunosuppression	Donor source	Graft status (event)
1	41	Hypertensive nephropathy	No dialysis	CsA + steroid	Living	Survival
2	25	Unknown (no pre-KT biopsy)	PD (12)	Tacrolimus + MMF+ steroid	Living	Survival (acute rejection)
3	31	Unknown	HD (3)	CsA + MMF + steroid	Living	Survival
4	28	Chronic glomerulonephritis	HD (51)	Tacrolimus + MMF + steroid	Living	Survival (acute rejection)
5	29	IgA nephropathy	HD (1)	CsA + steroid	Living	Fail

KT, kidney transplantation; CsA, cyclosporin A; PD, peritoneal dialysis; MMF, mycophenolate mofetil; HD, hemodialysis.

**Table 3:** Clinicopathologic characteristics of cervical cancer patients

Case No.	Age at the time of diagnosis	Histologic type	Pathologic diagnosis (or Clinical stage)	Treatment	Interval between KT and final diagnosis (months)	Patient status
1	41	Adenosquamous	Ib1	R/H with pelvic LN dissection	5.6	NED
2	27	LCNK, SCC	Ia1	T/H	27.7	NED
3	41	Adenocarcinoma	Ib2	R/H with pelvic LN dissection	124.9	NED
4	38	LCNK, SCC	Ib2	R/H with pelvic LN dissection	118.6	NED
5	40	LCNK, SCC	Ib1	R/H with pelvic LN dissection	136.8	NED

KT, kidney transplantation; R/H, radical hysterectomy; LN, lymph node; NED, no evidence of disease; LCNK, large cell nonkeratinizing; SCC, squamous cell carcinoma; T/H, total hysterectomy.

### Staining pattern analysis and HPV typing according to the HPV ISH of the cervical tissue

According to the HPV ISH of tissues from five patients, four patients (80%) tested positive for HPV. Punctate staining pattern was observed in three (75%) of these four patients, and one manifested the diffuse staining pattern. According to the HPV typing, high-risk HPV (types 16 or 58) was detected in three patients, and probably high-risk HPV (type 66) was detected in one patient (Table 4).

**Table 4:** Results of HPV typing and ISH

**Case No**.	HPV DNA chip	HPV high grade ISH
2	58, 70, 62	scattered, diffuse
3	66	punctate
4	16	punctate
5	16, 42	punctate

ISH, in situ hybridization.

## Discussion

Statistics from many developed countries show that the overall incidence of malignancy in patients who had received an organ transplant is about 2-4 times higher than in the general population, and that skin cancer and malignant lymphoma are the most common types of carcinoma in such patients. Moreover, the incidence of malignancy such as cervical carcinoma, malignant lymphoma, and Kaposi's sarcoma, which are attributable to a carcinogenic virus, are also higher than in the general population [4,16-18]. However, the types of malignancy that develop after an organ transplant reportedly vary by country and region. In South Korea, some studies reported skin cancer is most common in patients who had received an organ transplant [19,20] whereas other studies reported that cervical carcinoma is most common [21]. Although the incidence of malignant carcinomas such as cervical carcinoma, thyroid carcinoma, stomach carcinoma, and colorectal carcinoma is higher than in western countries, no national statistics are available. In this study, thyroid carcinoma is the most common and the annual incidence of malignancy that developed after an organ transplant was 418.2 out of 100,000 patients, about 1.7 times higher than the 246 in 100,000 South Korean women in the general population [22]. Cervical carcinoma develops after a renal transplant in 58.1 of 100,000 patients a year, 3.5 times higher than that in normal South Korean women (16.4 of 100,000 women)[22]. (Table 1).

Factors that increased the incidence of malignancy after the organ transplant were reported to be related to the age at the time of the organ transplant, the sex, the race, the type of immunosuppressant, a history of renal disease that required a transplant, and the period during which dialysis was performed prior to the renal transplant. Such factors, however, varied by study [4,7,23-25]. A close examination of the results of many studies showed that the incidence of malignancy after an organ transplant was higher in patients who were older at the time of the transplant [4,7,23-25] in men [4,7,23], in whites [4,7], in patients who used Tacrolimus as an immunosuppressant [7,23], in patients who received a renal transplant due to non-diabetic nepropathy [4,7,23], and in patients who underwent dialysis for three years or more before the organ transplant. In contrast, the risk of malignancy was lower in patients who were younger at the time of the organ transplant; in women, blacks, or Asians; in patients who used CD25 as an immunosuppressant [24]; in patients who received a renal transplant due to diabetic nephropathy; and in patients who underwent dialysis for a shorter period. In this study, as the patients with cervical carcinoma received a renal transplant at a young age (mean age: 30.8 years, 25-41 years) due to an unknown cause or a non-diabetic disease, the mean period of the dialysis before the renal transplant was as short as 13.4 months (1-51 months). Since there were only five patients, it was difficult to analyze the factors that can increase the incidence of cervical carcinoma after a renal transplant (Table 2).

Based on some studies, malignancy that developed in patients who received an organ transplant had a worse prognosis than that which developed in the general population [1,26,27], whereas others reported that it was not related to a worse prognosis [28,29]. In this study, surgical treatment was performed in five patients with cervical carcinoma (one patient, extrafascial hysterectomy only; four patients, type III radical hysterectomy and pelvic lymphadnectomy), but no additional chemotherapy or radiation therapy was performed. Because during the mean follow-up period of 93.2 months (18-190 months), no death or recurrence of disease was observed, it could not be concluded that the prognosis of cervical carcinoma that developed after the renal transplant was worse than that which developed in the general population (Table 3).

The incidence of HPV infection after a renal transplant was reported to be 15-45%[9,30-33]. It was also reported that an increase in opportunistic infection arising from the use of immunosuppressants increased the incidence of cervical intraepithelial neoplasia by 14 times compared to that of the age-adjusted control group [8]. Moreover, statistics from many developed countries indicate that the age-adjusted risk of cervical carcinoma developing after a renal transplant was 1.6-5.7 times that in the control group [4,16-18]. Early screening seems mandatory and screening methods have been extensively applied in patients who received an organ transplant. American and European societies of renal transplants recommend Papanicolaou test and pelvic examination. Due to the high incidence of HPV infection with low rate of cytologic alteration found at Pap test in renal transplant recipients, HPV screening test is also discussed as a primary screening method. However, no appropriate screening method has been established for these patients because little is known about the rate of progression to cervical carcinoma and the time to progression in renal transplant recipients. Large-scale randomized controlled trials on the effectiveness of the cytologic examination and HPV testing are needed [34-36].

The HPV ISH performed in this study is advantageous because it directly investigates whether or not the HPV DNA was integrated into the host's DNA; a diffuse staining pattern indicates episomal HPV; a punctate staining pattern in the nucleus indicates that integration occurred. Therefore, because it was reported that the integration of the HPV DNA plays an important role in the early stage of carcinogenesis, the appearance of the punctate staining pattern by the HPV ISH significantly increases as the cervical intraepithelial lesion progresses and in the case of invasive cervical carcinoma [13,37,38]. In this study, the experiment procedure was standardized using automatic equipment. Inform HPV III kit (Ventana Medical System), a detection system that has high sensitivity due to its increased signal strength, was used.^13 ^Eighty percent of the cervical lesions tested positive for HPV by the HPV ISH. Of these HPV-positive tissues, 75% had a punctate staining pattern, and 25%, a diffuse staining pattern (Table 4), which are consistent with the results of other studies [37,39].

The types of HPV that were detected in the cervical carcinoma were high-risk HPV (types 16 or 58) in three of four patients, and probably high-risk HPV (type 66) in one patient (Table 4). This suggests a direct relationship between high-risk HPV and infiltrating cervical carcinoma.

## Conclusions

In this study, the incidence of cervical carcinoma in patients who had received a renal transplant at the authors' hospital increased by 3.5 times compared to the general population. Based on this risk, it is suggested that appropriate screening tools be required for women who had received a renal transplant. Analysis of the clinical factors related to cervical carcinoma that develops after a renal transplant and analysis of the prognosis of such carcinoma are required, since there were only a few cases in this study. It is considered that collection and management of statistical data on patients who had received a renal transplant should be performed at the national level for more efficient analysis.

## Competing interests

The authors declare that they have no competing interests.

## Authors' contributions

STP contributed mainly in the design, literature review and writing of this work, while corresponding author CWL provided the idea, planned, edited and approved the written work. Both SYH and JSP gave valuable advices and edited the discussion. MJS worked on the clinical presentation. All authors read and approved the manuscript.
